# Supplementary material for: ZO-1 Guides Tight Junction Assembly and Epithelial Morphogenesis via Cytoskeletal Tension-Dependent and -Independent Functions
Source: Cells. 2022 Nov 25;11(23):3775. doi: 10.3390/cells11233775 (PMC9740252; doi:10.3390/cells11233775)
Supplement: Supplementary file 1 [file cells-11-03775-s001.zip › cells-1955666-supplementary.pdf]

SUPPLEMENTARY FIGURES

ZO-1 guides tight junction assembly and epithelial morphogenesis via cytoskeletal tension-dependent and -independent functions

Alexis J. Haas *et al.*

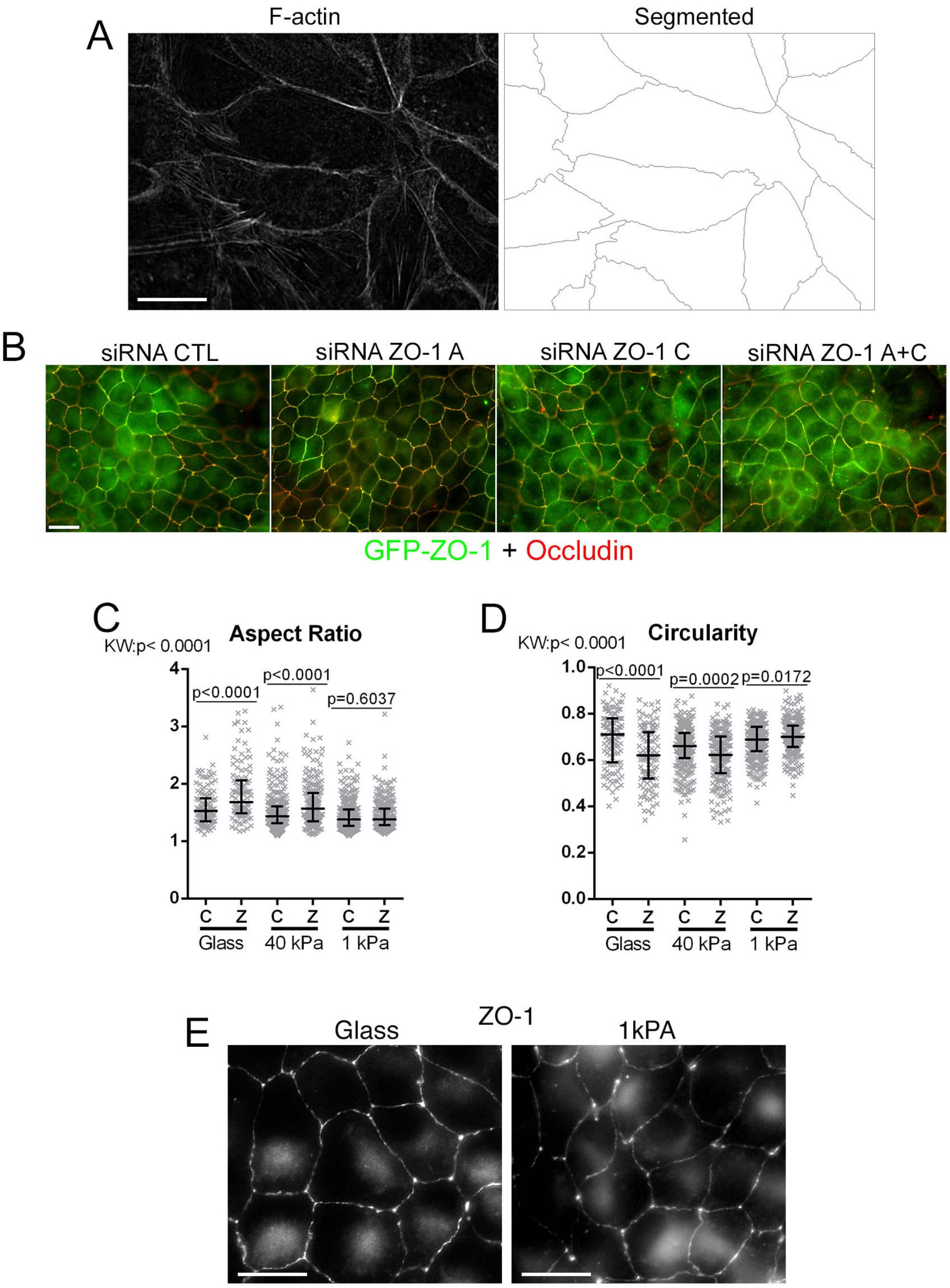

Figure S1. Expression of GFP-mZO-1 and soft ECM rescue the ZO-1 depletion phenotype. **A** Example of image segmentation used for the morphometric analysis. Shown is an F-actin staining and the corresponding image mask. **B** GFP-mZO-1 expressing MDCK cells were transfected with siRNAs as indicated and then plated on glass followed by immunostaining for occludin. Images show overlays of GFP and occludin images. **C, D** Wild-type MDCK cells transfected with control (c) or pooled siRNAs against ZO-1 (z) were seeded on glass or hydrogels (40 or 1kPa). Junctional immunostaining was then performed, and the resulting images were used to segment individual cells and perform a morphometric analysis of aspect ratios (C) and circularity (D). Data points represent analysed cells. **E** MDCK cells plated on glass or 1kPa hydrogels were processed for epifluorescence microscopy using an antibody against ZO-1. Magnification bar, 20 μm.

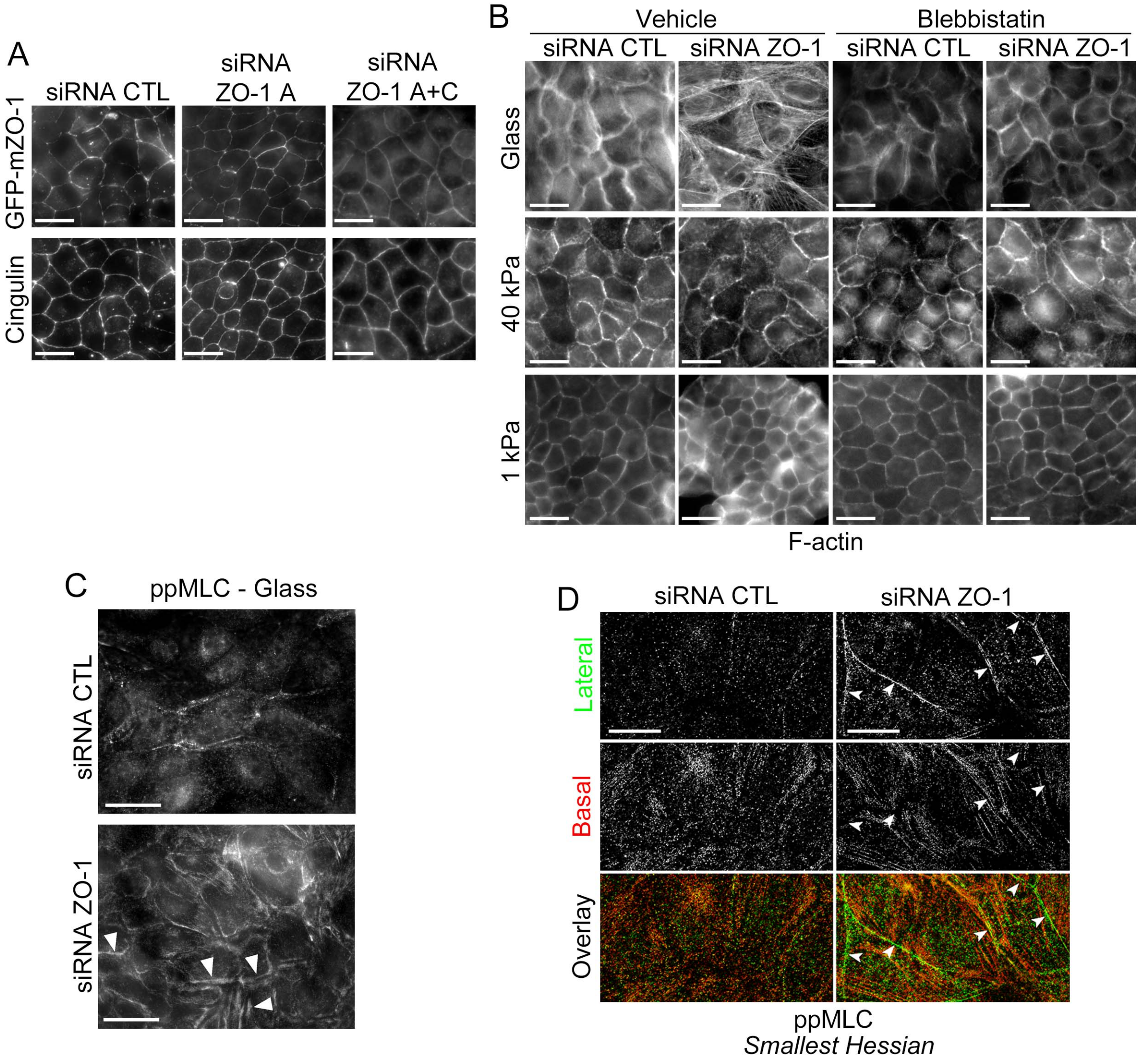

Figure S2. Junction formation rescue and actomyosin remodelling upon ZO-1 depletion. **A** The GFP-mZO-1 cell line was transfected with siRNAs as indicated before immunolabelling cingulin and imaging. **B-D** siRNA transfected MDCK cells were seeded on glass, or 40 kPa or 1 kPa hydrogels. After cell fixation, cells were labelled using phalloidin to visualize F-actin (B) or ppMLC (C and D, cells gown on glass). In panel C, epifluorescence images from the base of the cells are shown, and thick actomyosin bundles are marked with arrowheads. In panel D, microscopy images were processed using the smallest Hessian differentiation operation to enhance the visual discrimination between basal and lateral features of actomyosin. Magnification bars, 20 μm.

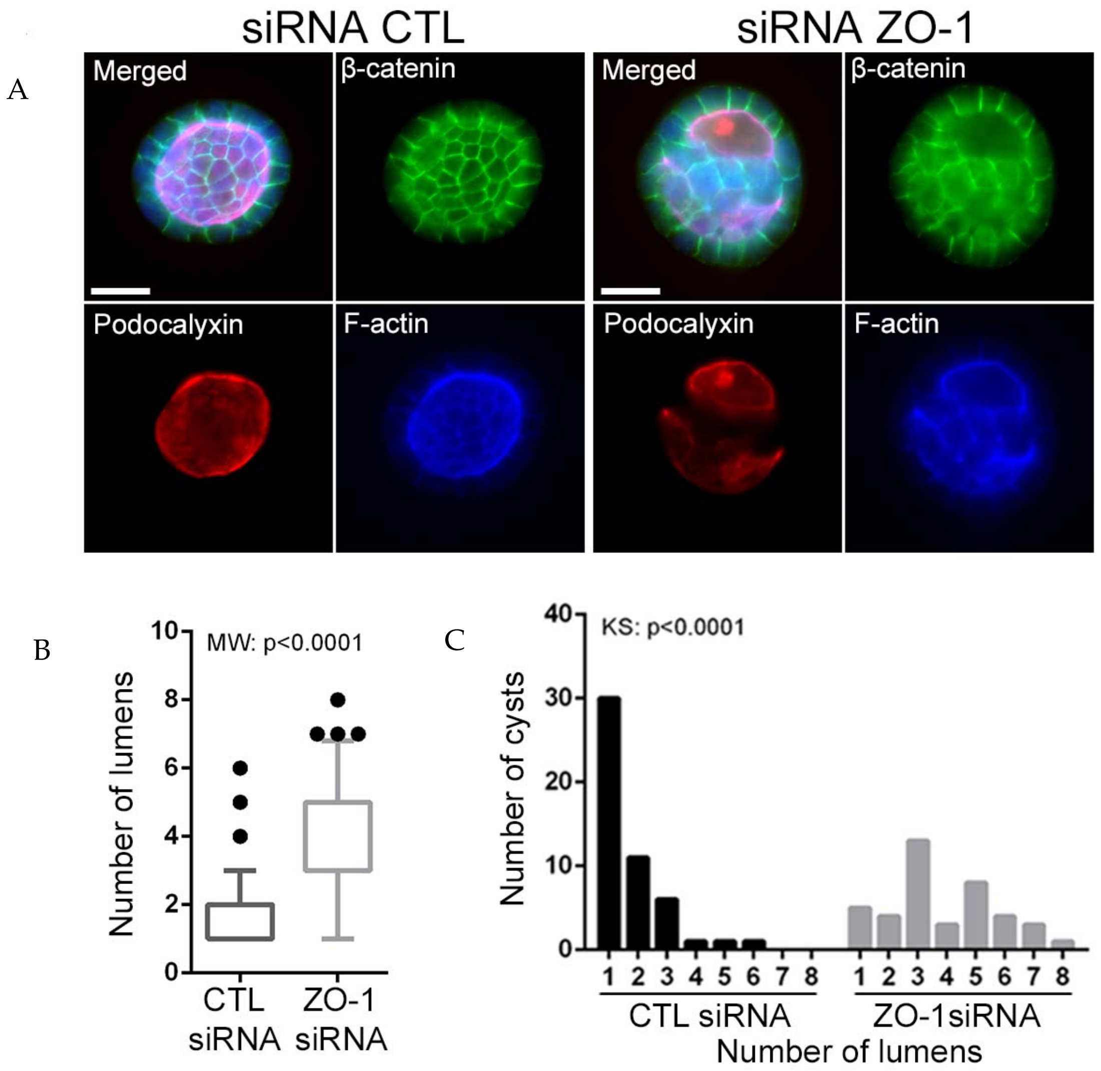

Figure S3. ZO-1 deficiency disrupts 3D morphogenesis.
(A) siRNA transfected MDCK cells were seeded in a matrix consisting of Matrigel and collagen I to form 3D spheroids. (B) After fixation the cysts were stained as indicated, the number of lumens per cyst was manually counted, (c) and the distribution of the cysts population was plotted over the number of lumens per cyst counted in each condition. Magnification bars, 20 µm.

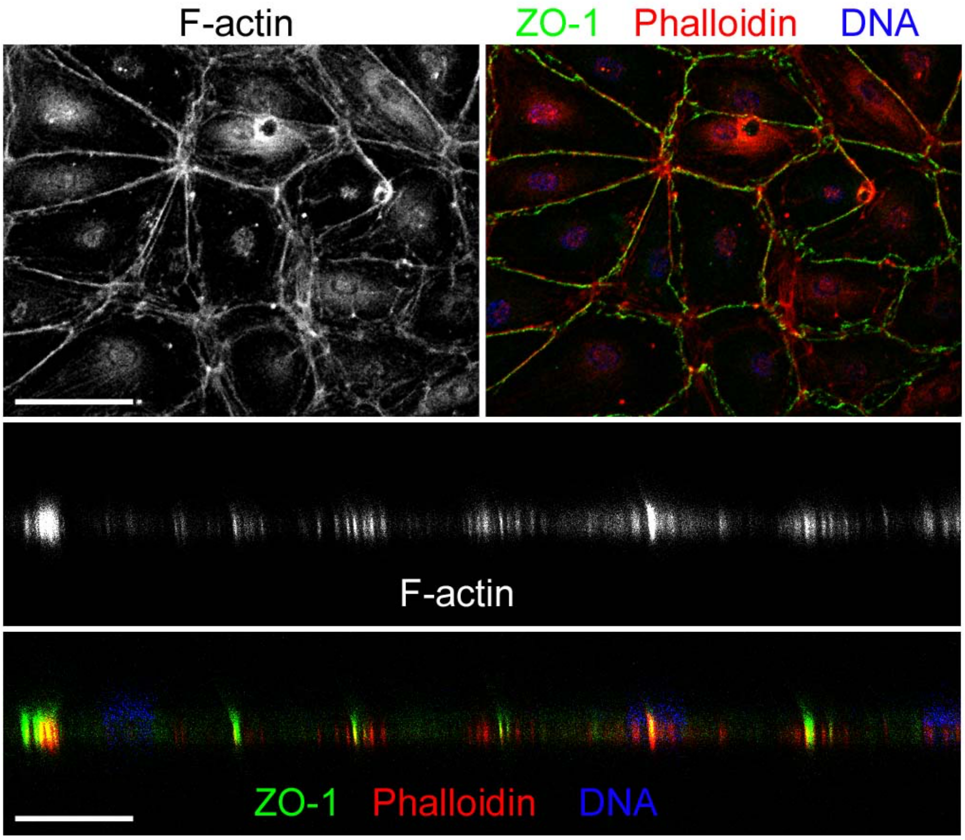

Figure S4. F-actin distribution in primary microvascular endothelial cells.
Endothelial cells were seeded on Matrigel-coated glass and immunostained for ZO-1. F-actin was labelled with fluorescent phalloidin and nuclei with Hoechst dye. Cells were visualized in xy (top panel) and in xz orthogonal cross-sections (middle and bottom panels). Magnification bars, 20 μm.

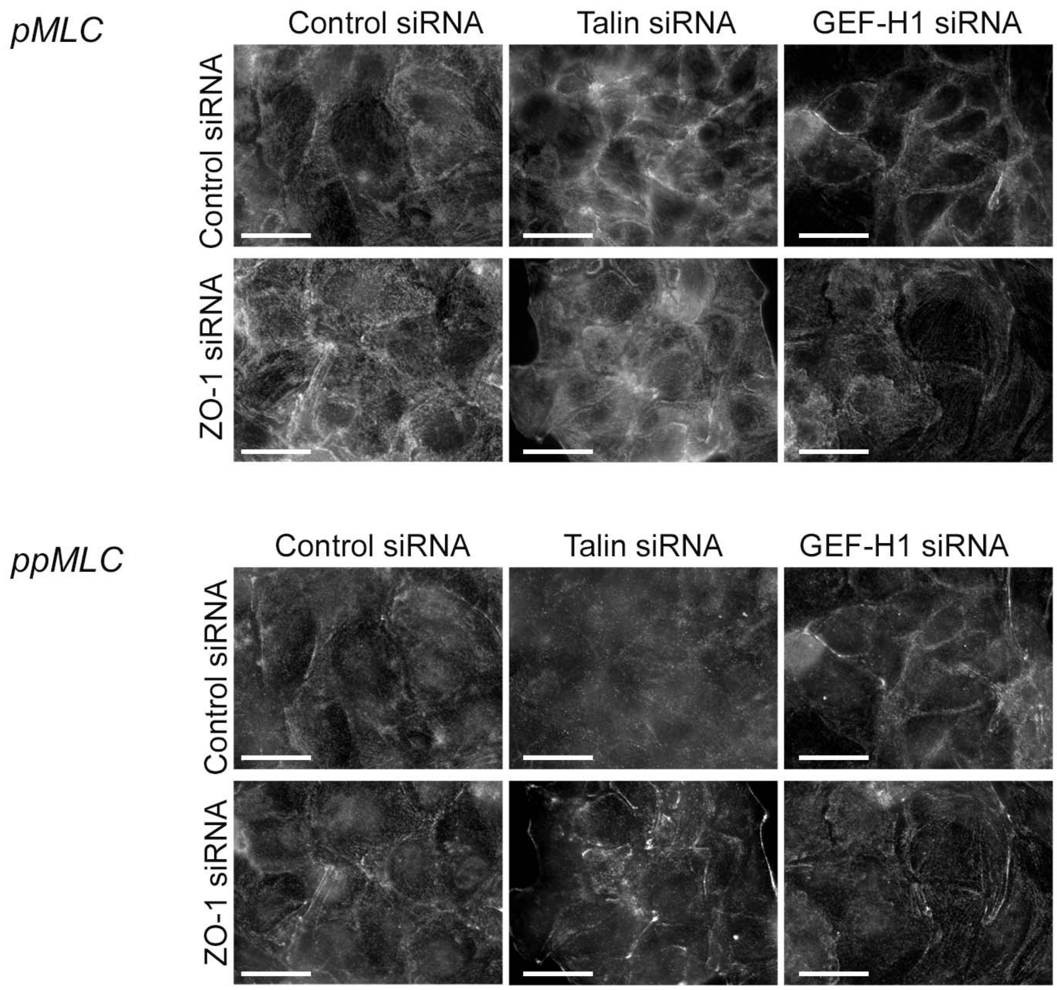

Figure S5. Staining for phosphorylated MLC in siRNA transfected cells.
MDCK cells transfected with siRNAs as indicated were plated on Matrigel-coated glass and then stained and processed for immunofluorescence with antibodies against single (pMLC) and double (ppMLC) phosphorylated MLC. Shown are epifluorescence images derived from the basal part of the cells. Magnification bars, 20 μm.

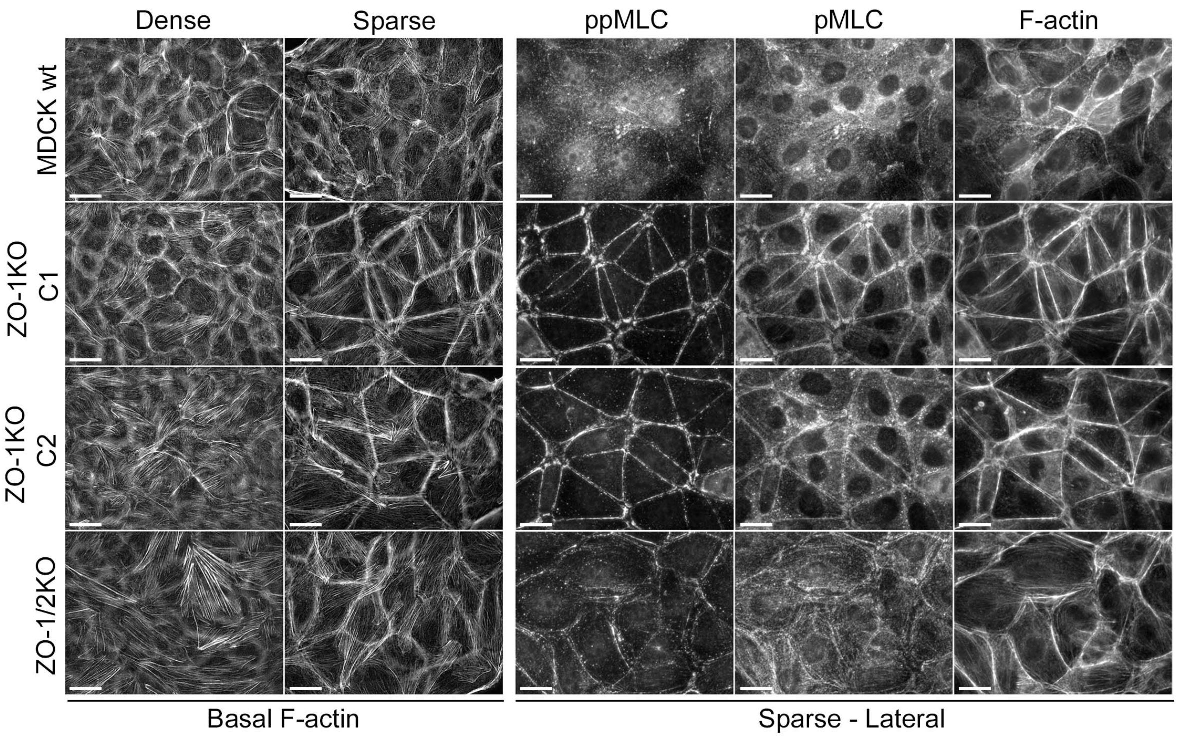

Figure S6. ZO-1 knockout stimulates actomyosin remodelling.
Wild-type and knockout MDCK cells were seeded on glass or hydrogels (40 kPa and 1 kPa) before fluorescently staining F-actin, pMLC and ppMLC. The most in-focus basal slices from acquired z-stacks were enhanced to visualize stress fibres in sparse and dense areas with the F-actin staining (left panels). Images showing lateral focal planes of sparse areas are shown for pMLC, ppMLC and F-actin (right panels). Magnification bars, 20 μm.

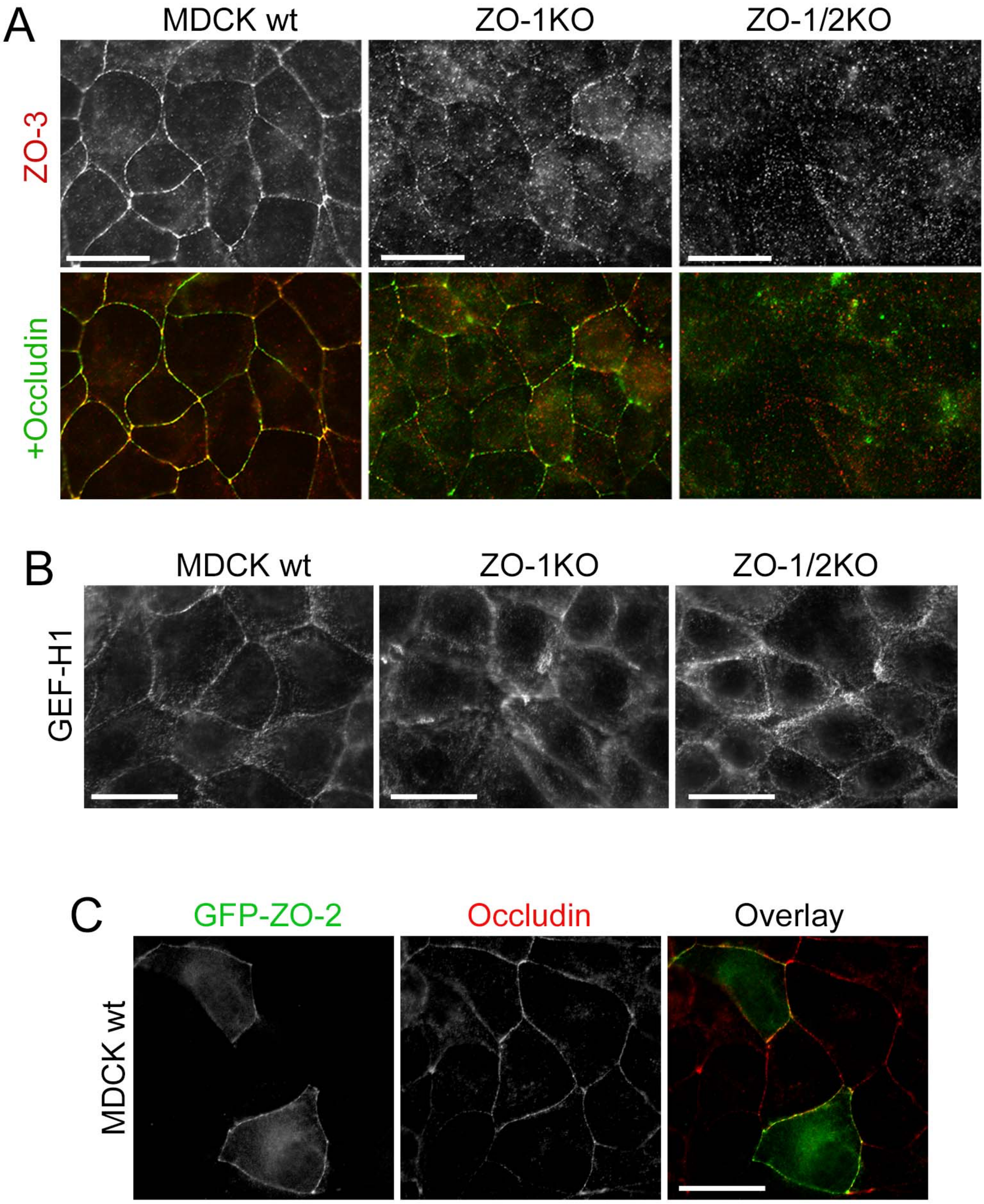

Figure S7. TJ assembly analysed in highly confluent cells.
A, **B** Wild-type and knockout MDCK cells grown for 5 days were fluorescently labelled with antibodies against the indicated proteins and imaged by bright field microscopy. C Wild-type MDCK cells transfected with GFP-ZO-2 were counterstained for occludin and imaged by epifluorescence microscopy. Magnification bars, 20 µm.

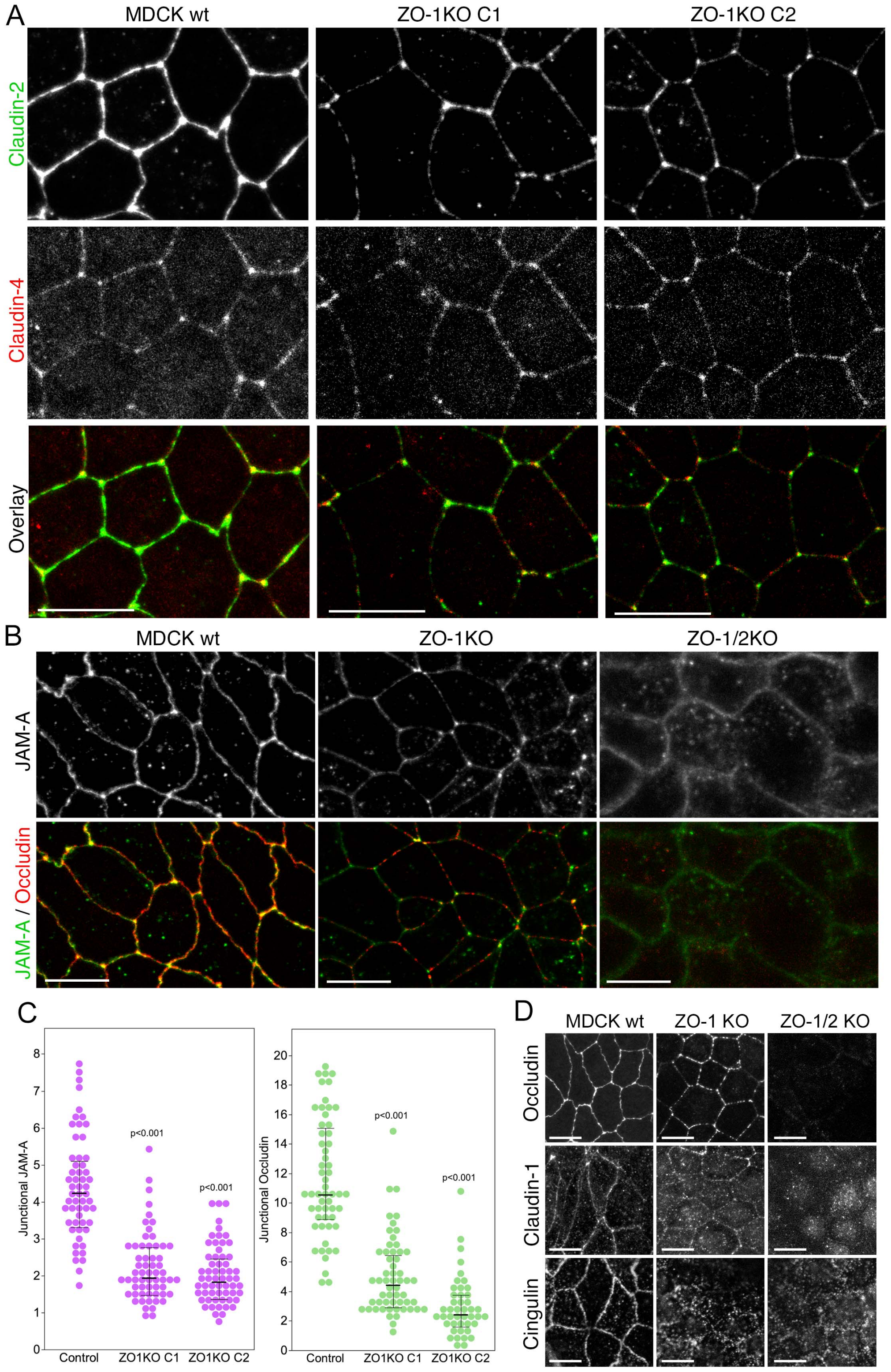

Figure S8. TJ assembly analysed in filter-grown cells.
(A) Wild-type and knockout MDCK cells were grown on filters prior to analysis by immunofluorescence and confocal microscopy using antibodies against the TJ proteins indicated. (B) All confocal XY sections are maximum intensity projections of all sections containing junctional staining. The fluorescence intensities of JAM-A and occludin at cell junctions were quantified in z-sections (C). Magnification bars, 20 μm. (D)

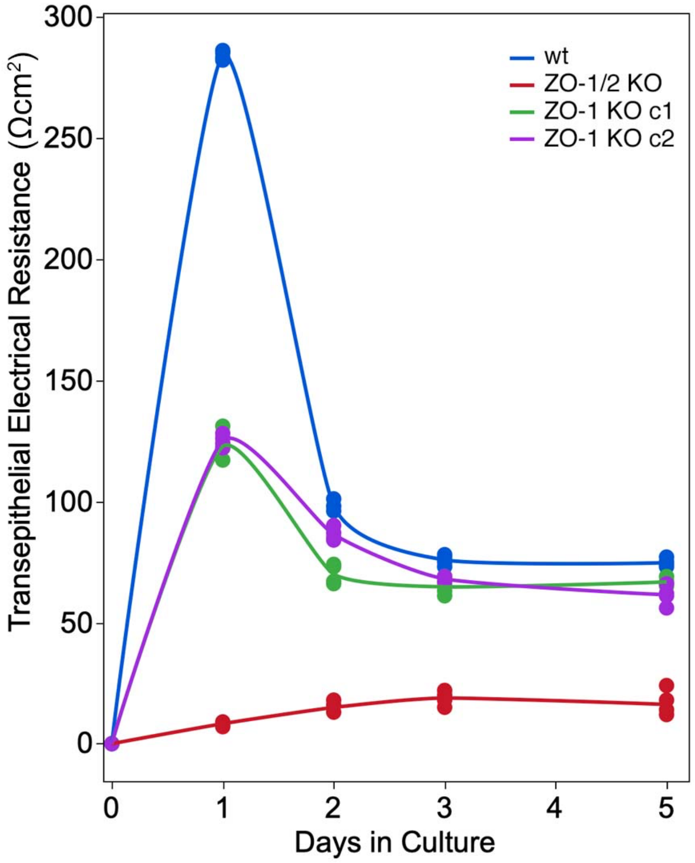

Figure S9. TER development of ZO-1 KO cell lines.
The indicated cell lines were plated on filter inserts and TER was measured at the indicated time points until an equilibrium was reached. Shown are data points derived from individual filter cultures.
